# Prevalence of Intestinal Parasites in Young Quichua Children in the Highlands of Rural Ecuador

**Published:** 2007-12

**Authors:** Kathryn H. Jacobsen, Priscila S. Ribeiro, Bradley K. Quist, Bruce V. Rydbeck

**Affiliations:** 1 Calvin College, Grand Rapids, Michigan, USA and Department of Global and Community Health, George Mason University, Fairfax, Virginia, USA; 2 University of Michigan Medical School, Ann Arbor, Michigan, USA; 3 Health Care Division, HCJB World Radio, Quito, Ecuador; 4 Community Development Vozandes, Health Care Division, HCJB World Radio, Quito, Ecuador

**Keywords:** Ascariasis, Child, Child, Preschool, Cryptosporidiosis, Entamoebiasis, Epidemiology, Giardiasis, Parasites, Intestinal diseases, Parasitic, Risk factors, Ecuador

## Abstract

The prevalence of intestinal parasites in young Quichua children was assessed in 20 rural communities in the highlands of Ecuador in August 2005. The caregivers of 293 children aged 12–60 months were interviewed about the status of child health, household socioeconomic and environmental factors, and water-use practices and were requested to collect a faecal sample from the study child. Two hundred three (69.3%) of the 293 children provided faecal samples that were tested for parasites. The overall prevalences of infection for specific agents were *Entamoeba histolytica* or *dispar* 57.1%, *Ascaris lumbricoides* 35.5%, *Entamoeba coli* 34.0%, *Giardia intestinalis* (*lamblia*) 21.1%, *Hymenolepis nana* 11.3%, *Cryptosporidium parvum* 8.9%, *Chilomastix mesnili* 1.7%, *Hymenolepis diminuta* 1.0%, *Strongyloides stercoralis* 0.7%, and *Trichuris trichiura* 0.5%. The prevalence of parasites increased with age. Water storage, water treatment, consistent latrine-use, and participation in a community-based clean water project were not strongly associated with the prevalence of intestinal parasites, although having dirt floors was a risk factor for infection with *E. histolytica* or *dispar* and *G. intestinalis*.

## INTRODUCTION

The goal of this study was to determine the prevalence of various parasitic infections in young children in rural Quichua communities in Ecuador. The study was also aimed at assessing the relationship of latrine-use, water storage, water treatment, and community-based clean water projects that protected sources of drinking-water with illness and prevalence of intestinal parasites in children.

Several other epidemiological studies have assessed the prevalence of intestinal parasites in Ecuador or other parts of South America (1–10), but few have focused on rural areas, preschool children, the highland areas of the Andes, or Quichua populations. In addition to focusing on an under-studied population, we also tested for a greater variety of parasites than most previous studies. Stool samples were tested for several types of protozoans, such as *Entamoeba histolytica/Entamoeba dispar, Entamoeba coli, Giardia intestinalis* (*lamblia*), *Chilomastix mesnili*, and *Cryptosporidium parvum*, roundworms, such as *Ascaris lumbricoides* and *Strongyloides stercoralis*, tapeworms, such as *Hymenolepis nana*, also known as dwarf tapeworm, and *Hymenolepis diminuta*, and whipworm, *Trichuris trichiura*. Most of these agents are pathogenic, except *E. dispar, Eschericia coli*, and *C. mesnili*. Most of these parasitic agents have multiple possible transmission routes, including contact with contaminated food, water, soil, or excrement, so the availability of clean drinking-water, an adequate volume of water for hygiene, and a sanitation system that properly disposes of human excrement may contribute to decreasing the incidence of these infections.

## MATERIALS AND METHODS

### Community participants

Ten rural Quichua communities in the highlands of Chimborazo province in central Ecuador that had completed a clean water project in cooperation with a non-governmental community development organization at least one year prior to the start of the study were selected for inclusion in the study. The clean water projects consisted of three main components: (a) a protected water source, usually a spring, (b) a water-distribution system with protected water storage and buried piping that provides water to a spigot outside each home, and (c) a latrine for each household. Ten neighbouring communities with similar socioeconomic and geographic characteristics that had not yet participated in a clean water project were selected as control communities. All communities were more than 2,750 metres above the sea level. A member of the community development organization that facilitated the water projects visited each of the 20 communities in June 2005 to invite participation and again one or two day(s) prior to the survey day in August 2005 to prepare for the site visit. Letters reminding the community of the date of the community survey were sent to the elected president of each community in June and again two weeks before the study.

### Child study population

We asked the elected president of each community to complete a community profile and provide a list of age-eligible children in the community before the study period. All households in each community with a child aged 12-60 months were invited to participate. If more than one child in the household was in the eligible age range, the youngest eligible child was selected for inclusion. Most communities listed 10–20 children aged 12–60 months. We were unable to determine an exact participation rate because not all communities provided a list of eligible children, and some children on the list were siblings, so they were not eligible for inclusion. We believe that we had a high participation rate because, in most communities that provided a list of children, all or nearly all the listed children participated in the study or were known to be a sibling of a child who participated. Free parasite testing and treatment and a physical examination by a physician were offered to all the children in the community under the age of 18 years, regardless of whether the household was participating in the study.

### Questionnaire data

The questionnaire was pretested in June 2005 in two communities that were not included in the study. Data were collected in the 20 participating communities in August 2005. After explaining the study process both orally and in writing, informed consent was obtained from parents or guardians of minors, following the protocol that was approved by the Institutional Review Board of the Calvin College, Michigan, USA. Interviews were conducted in Spanish, with the assistance of a Quichua translator when needed. Questions about the status of child health, socioeconomic and environmental characteristics of households, and water-use practices were answered by a family member. The health-history section asked if the child had a fever, diarrhoea, vomiting, respiratory illness, or other illnesses in the past week or past month, if the child had an eye infection or skin infection in the past six months, and if the child had been treated for parasitic infection within the last six months. The caregiver was asked to collect a stool sample from the child, if the child was able to provide a sample during the site visit.

### Laboratory techniques

The faecal samples were tested in a hospital laboratory in Chimborazo province on the day of collection for the presence and intensity of *E. histolytica* or *E. dispar* cysts, *A. lumbricoides* eggs, *E. coli* cysts, *G. intestinalis* (*lamblia*) cysts, *H. nana* eggs, *C. parvum* oocysts and trophozoites, *C. mesnili* cysts and trophozoites, *H. diminuta* eggs, *S. stercoralis* larva, and *T. trichiura* eggs. Samples were collected in plastic containers in the morning, stored in an insulated container, and transported to the laboratory within a few hours of collection. Prior to transportation, part of the sample was dissolved in a 10-mL fixative solution (formaldehyde 10%, Triton X-100 0.1%, NaCl 0.85%, pH 7.0). A formalin-ether concentration method was performed on these samples, and they were examined using light microscopy. The remainder of the sample was not preserved and was used for preparing fresh slides in a physiological saline solution to look for nematode ova and protozoan cysts and trophozoites. A Lugol's iodine water solution (KI 2%, I 1%) was used for wet-mount preparations. In addition, to detect oocysts of *Cryptosporidium* spp., a thin film (smear) of fresh faeces on a clean glass slide was prepared and dried at room temperature in the field and was then examined by microscopy after conducting a Kinyoun's carbol fuchsin modified acid-fast stain.

### Data analysis

Survey and laboratory data were entered into a spreadsheet using the Epi Info software (version 3.3.2). The SPSS software (version 12.0) was used for statistical analysis, and the significance level was set at α=0.05.

## RESULTS

### Sample population

In total, 149 (50.9%) of the 293 children were from the intervention (community-based clean water project) communities, and 144 (49.1%) were from the control communities. There were no differences between children from intervention and control communities in age distribution, house construction (roof, walls, floors, and number of rooms), household size, parental education, ownership of animals, cooking-location, reported consistency of latrine-use, or reported treatment for parasites in the past six months. The control community residents were more likely to have a Centro de Salud (health clinic) in the community (44.4% vs 7.4%, p<0.001). The control community residents were also more likely than the intervention community residents to store water in small containers, such as a bucket, a pail, or a pot (32.9% vs 10.2%, p<0.001) and large tanks (38.5% vs 18.4%, p<0.001), but reported the same rate of boiling drinking-water (24.1%).

### Faecal sample donation

One hundred twelve (75.2%) of the 149 children from the intervention communities and 91 (63.2%) of the 144 children from the control communities provided stool samples. In total, 203 (69.3%) of the 293 children provided a stool sample, of which 55.2% were from the intervention communities and 44.8% were from the control communities. There were no statistically significant differences between the 203 donors and the 90 non-donors in age distribution, having a clinic in the community, reported treatment for parasites in the past six months, or reported diarrhoea in the past week or month, any illness in the past week or month, or eye infection or skin infection in the past six months. Donors from the intervention communities reported a higher rate of illness in the past week (84.8% vs 64.8%, p=0.031) or month (96.4% vs 82.4%, p=0.016) and skin infection in the past six months (48.2% vs 24.4%, p=0.034) than non-donors. There was no difference in reported diarrhoea in the past week (32.7% vs 24.2%, p=0.840) or month (57.5% vs 54.4%, p=0.254) or latrine-use. In the control communities, there were no statistically significant differences in reported illness, but donors were more likely to always use a latrine (p<0.001).

### Intestinal parasites

Of all the 203 samples donated, 85.7% had at least one of the 10 parasites tested for, and 63.4% were infected with two or more of the parasites. The overall prevalence of parasite infection for specific agents were *E. histolytica* or *E. dispar* 57.1%, *A. lumbricoides* 35.5%, *E. coli* 34.0%, *G. intestinalis* (*lamblia*) 21.1%, *H. nana* 11.3%, *C. parvum* 8.9%, *C. mesnili* 1.7%, *H. diminuta* 1.0%, *S. stercoralis* 0.7%, and *T. trichiura* 0.5%. 78.3% had at least one protozoan infection, and 42.4% had at least one helminth infection. There were no differences in reported illness (diarrhoea, fever, respiratory infection, vomiting, or other) by intestinal parasitic infection, except for slightly-increased levels of reported diarrhoea in the past month in children with *E. histolytica* or *dispar*-associated infection (65.2% vs 49.6%, p=0.032).

### Age

**Fig. 1:**
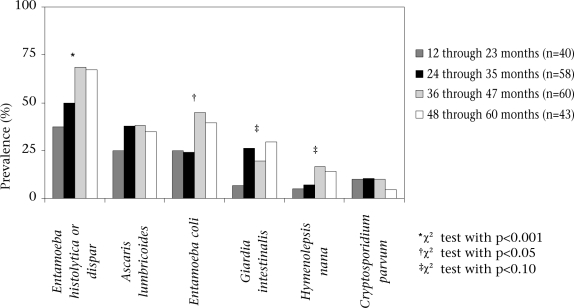
Intestinal parasites, by age

The prevalence of intestinal parasites increased with age. Figure 1 shows laboratory results by age (12–23 months, 24–35 months, 36-47 months, and 48-60 months) for faecal sample donors. Older children were more likely to have at least one of the 10 parasites tested for (65.0%, 84.5%, 95.0%, 93.0%, p<0.001) and more than one of the 10 parasites tested for (35.0%, 55.2%, 71.1%, 65.1%, p=0.002). Significant trends were seen for *E. histolytica* or *dispar* (37.5%, 50.0%, 68.3%, 67.4%, p<0.001) and *E. coli* (25.0%, 24.2%, 45.0%, 39.5%, p=0.030). Less significant trends were observed for *H. nana* (5.0%, 6.9%, 16.7%, 14.0%, p=0.068) and *G. intestinalis* (6.5%, 26.0%, 19.6%, 29.3%, p=0.070). The trends for *A. lumbricoides* (25.0%, 37.9%, 38.3%, 34.9%, p=0.386) and *C. parvum* (10.0%, 10.3%, 10.0%, 4.7%, p=0.407) were not significant. Younger children were significantly more likely to have diarrhoea in the past week (39.4%, 34.5%, 19.5%, 13.8%, p=0.013) or month (67.6%, 66.3%, 20.3%, 15.8%, p<0.001). There was no difference in the proportion of children in each age-group living in the intervention communities (50.0%, 67.2%, 53.3%, 44.2%, p=0.275).

### Clean water projects

**Fig. 2:**
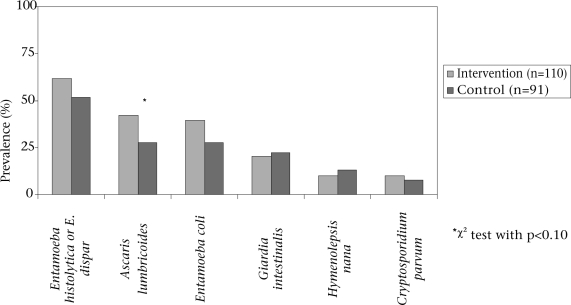
Intestinal parasites by status of clean water project

The households in the intervention and control communities had similar prevalence rates of intestinal parasites (Fig. 2). Controlling for age (in years) and the presence of a health clinic in the community in a logistic regression, the only potentially-significant difference for a particular agent was for *A. lumbricoides*, which had a slightly higher prevalence in the intervention communities than in the control communities (42.0% vs 27.5%, p=0.075). The intervention communities reported more illness in the past month (93.2% vs 84.0%, p=0.007), eye infection in the past six months (31.5% vs 21.0%, p=0.009), and skin infection in the past six months (43.0% vs 25.7%, p=0.012) than the control communities. There were no differences in reports of any illness in the past week or diarrhoea in the past month.

### Potential risk factors

**Fig. 3:**
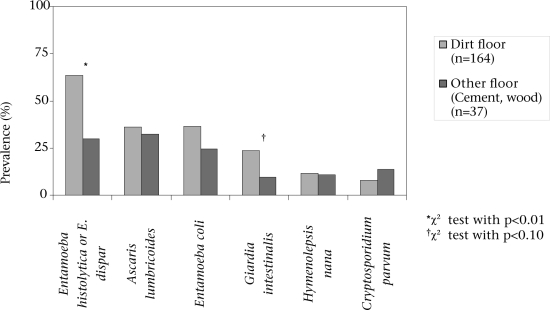
Intestinal parasites by flooring material

After adjusting for age and the presence of a clinic in the community, there was no association between storage of water, water treatment, or consistency of latrine-use and the outcomes relating to intestinal parasites or illness. Compared to households with cement or wood floors (18.4% of participating households), households with dirt floors (81.6% of households) had higher rates of *E. histolytica/E. dispar* (63.4% vs 29.7%, p<0.001) and *G. intestinalis* (23.8% vs 9.7%, p=0.075) (Fig. 3). Nearly all the houses had walls constructed with adobe, block, or brick, so wall material was not predictive of intestinal parasites or illness. The most common roofing materials were asbestos (43.9%), zinc (30.6%), and straw (10.3%), and there were no significant differences in the prevalence of intestinal parasites or reported illness based on roofing material.

## DISCUSSION

### Comparison to previous studies

Our study assessed the prevalence of parasites in an under-studied population group: young Quichua children in the highlands of rural Ecuador. We found a prevalence of intestinal parasites greater than that reported in most other similar studies from Ecuador (1–5) and other parts of South America (6–10). The only exception to the generally high levels of intestinal parasites we observed was a lower prevalence of whipworm than that found in other studies, which might be related to the high altitude (11). Protozoan infections in our study were more common than helminthic infections. The increased prevalence we observed likely reflects our rural study population (rather than an urban or suburban population) and focus on young children.

We found an 8.9% prevalence of *Cryptosporidium* oocysts. A few other studies from South America have tested for *Cryptosporidium* in children. The prevalence of *Cryptosporidium* in our study was similar to that found by Guderian *et al.* in Ecuadorian children hospitalized with diarrhoea (12). That study found no *Cryptosporidium* oocytes in children without diarrhoea, but only seven of 18 children with *Cryptosporidium*-associated infection in our study reported diarrhoea in the past week.

Our finding that older children aged 3–4 years have a higher prevalence of parasites than younger children aged 1–2 year(s) is consistent with findings of other studies (1,4,5,7), as is the report of lower incidence of diarrhoea in older children (13,14).

### Sample limitations

There were several potential limitations to our sample. First, although we attempted to enumerate the total number of all children aged 12-60 months by asking each community to provide a list of children before the survey was conducted, not all communities provided lists. Some eligible children were not available because their families were in the city for a school break or market, or their families were working in the fields until evening. Second, all residents of communities that participated in clean water projects were assumed to have participated fully, especially in their consistent use of a protected source of drinking-water even when other sources of water were available to households. We also assumed that all the intervention communities had similar experiences with their clean water projects. There were no statistically significant differences in reported illness or parasitic infection between intervention communities with older water systems (installed during or before 2001) and newer water systems (installed after 2001), but there may have been other ways of classifying these communities that we neglected to assess.

Third, parents may have inaccurately recalled illnesses of their children in the past week and month, although we do not believe that recall differed based on participation in a clean water project or other factors. A previous study in Ecuador observed a good agreement between reported diarrhoea in the past two weeks and the prevalence of serum antibody to rotavirus and supports the reliability of diarrhoea recall (13). We believe that we can expect similarly good recall. Fourth, donors from the intervention communities reported more illness than non-donors. This may have had a limited effect on the parasitological results since, among donors, intestinal parasites were not strongly associated with illness, but could mean that the prevalence in the intervention communities shown in the Figure 2 is slightly higher than the true rate.

### Risk factors

Water-related risk factors, such as consistent latrine-use, participation in a community-based clean water project, and storage and treatment of water were not associated with significant differences in the prevalence of intestinal parasites. We did find that dirt floors were associated with the greater prevalence of parasites, which is not surprising since most agents we tested for are transmitted primarily through faecally-contaminated soil and food.

We had expected lower rates of illness and intestinal parasites in the intervention communities because of the widely-known benefits of improved water supply and sanitation (15). Instead, we found no differences in the prevalence of intestinal parasites or reported diarrhoea, and the clean water project communities reported higher rates of respiratory illness and fever in the past month and skin infection in the past six months. Since the intervention communities were not selected for participation in a community water project based on greater need than the control communities, the possible reasons for the differences in reported illness are unclear. Parents in the intervention communities may be over-reporting illnesses or be more attuned to the health problems of their children than parents in the control communities, but we believe that the laboratory findings are valid. A likely reason for the similarities in the incidence of diarrhoea and the prevalence of parasites in the intervention and control communities is that there was no difference in the reported frequency of bathing and hand-washing in the intervention and control communities. The installation of sources of clean water and sanitation facilities does not automatically result in changes in hygiene behaviour and sanitation practices, so adding an additional health-education component to the protocol for community water projects conducted in this region might lead to a reduction in diarrhoea and intestinal parasites.

Further studies should also seek to determine, if possible, the separate health effects of increased quality of water, increased quantity of water, closer proximity of households to a water source, and improved reliability of the water system. While the improved quality of water should reduce the incidence of diarrhoea, it may have a relatively little impact on the prevalence of the parasites we tested for since only *Cryptosporidium* and *Giardia* have water as a primary mode of transmission. Increased quantity of water and proximity to a source of water could increase the frequency of bathing and hand-washing and lead to a decrease in the prevalence of parasites by reducing the rate of transfer of parasite eggs to the mouths of children. Reliability may influence susceptibility to infection and alter water-storage practices. Understanding the mechanisms through which water development impacts health behaviour and health outcomes will enable health educators to focus their message to communities.
